# Supplementary material for: Suppressor mutations in Rpf2–Rrs1 or Rpl5 bypass the Cgr1 function for pre-ribosomal 5S RNP-rotation
Source: Nat Commun. 2018 Oct 5;9:4094. doi: 10.1038/s41467-018-06660-w (PMC6173701; doi:10.1038/s41467-018-06660-w)
Supplement: Supplementary file 1 — Supplementary Information [file 41467_2018_6660_MOESM1_ESM.pdf]

Supplementary Information

Suppressor mutations in Rpf2–Rrs1 or Rpl5 bypass the Cgr1 function for pre-ribosomal 5S RNP-rotation

Thoms et al.

Supplementary Figures 1-10

Supplementary Tables 1-3

Supplementary References

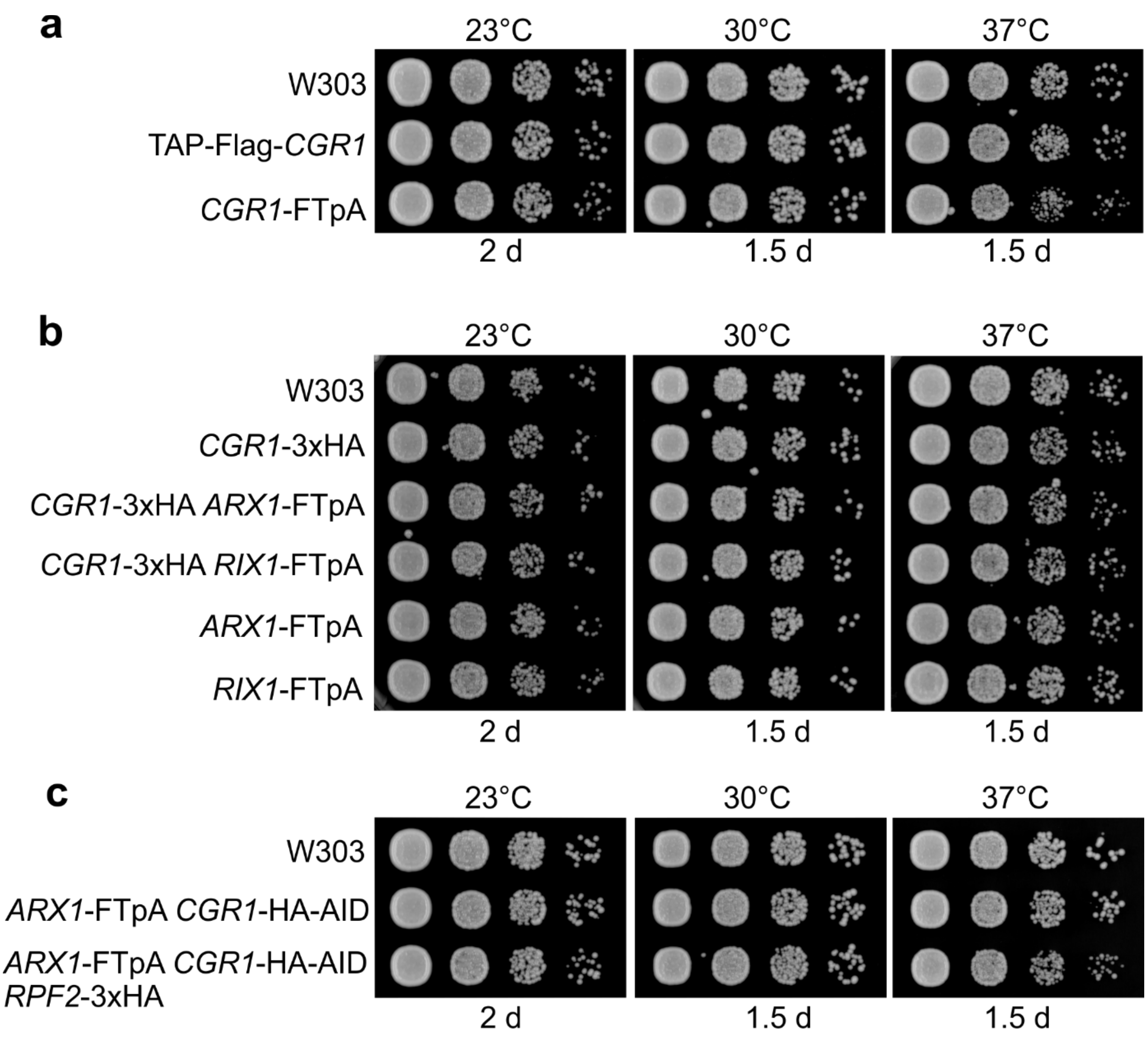

Supplementary Fig. 1 | Strains expressing chromosomal *cgr1* fusions display growth rates comparable to wild-type. a–c, Cells of the wild-type W303 strain[1] and of strains expressing the indicated N- or C-terminally tagged *cgr1* alleles used in this study were spotted in 10-fold serial dilutions on YPD plates and colony growth was monitored at the indicated temperatures and times.

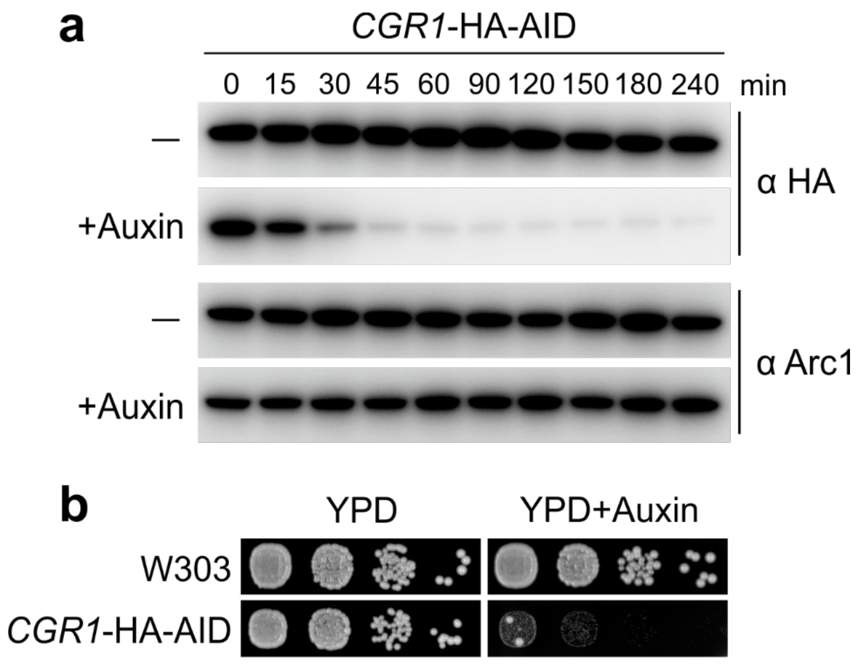

Supplementary Fig. 2 | Cgr1–HA–AID is efficiently degraded in the presence of auxin. a, Efficient auxin-induced Cgr1 depletion in *C*gr1–HA–AID cells. Logarithmically growing *CGR1*–HA–AID cells were treated at time point t_0 with 0.5 mM auxin (+) or not treated (−), and the level of Cgr1 degradation was assessed over 240 min by western blot analysis of whole cell lysates, using an anti-HA antibody to detect Cgr1. Equal sample loading was controlled by western blot analysis using anti-Arc1 antibodies. **b**, Growth of wild-type and Cgr1-depleted cells. Cells of the wild-type W303 strain or the *CGR1*–HA–AID degron strain were spotted in 10-fold serial dilutions on YPD plates in the absence or presence of 0.5 mM auxin (3-indoleacetic acid). Colony growth at 30 °C was monitored after 36 h incubation.

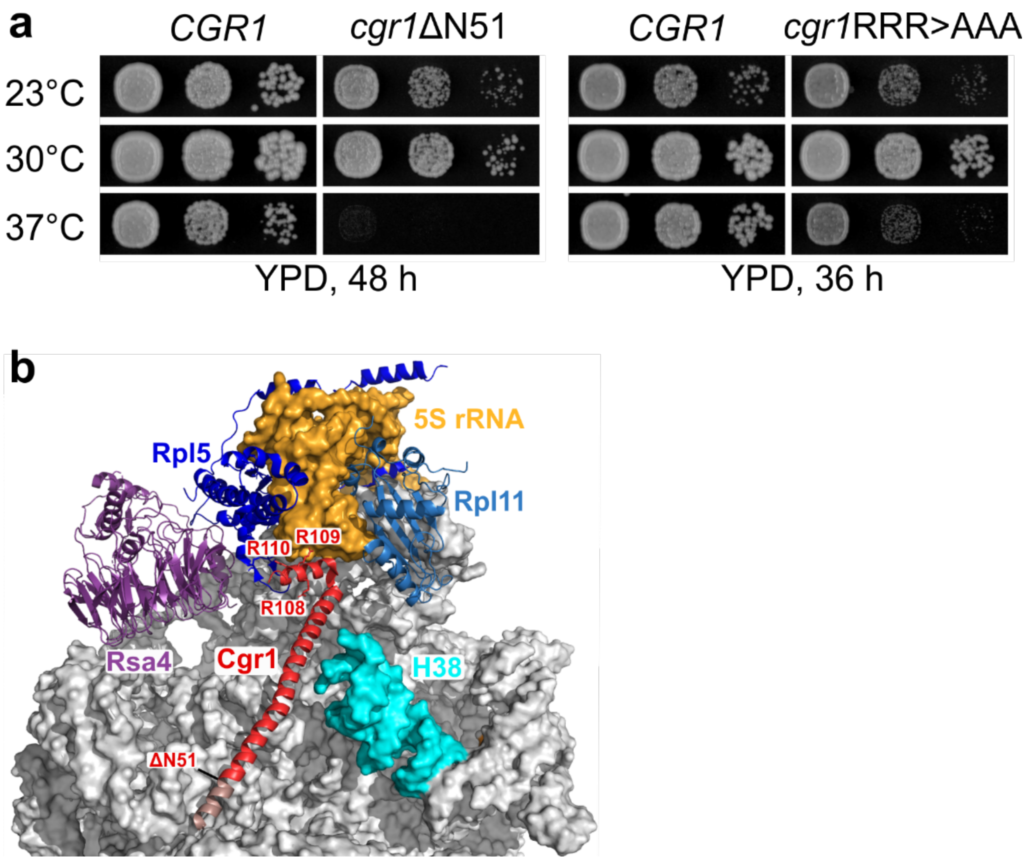

Supplementary Fig. 3 | Growth analysis of wild-type *CGR1* and mutant *cgr1*ΔN51 and *cgr1*R108A R109A R110A (RRR>AAA) cells. a, Shuffle strain *cgr1*Δ was transformed with plasmids carrying *CGR1* wild-type or the indicated mutant alleles. Transformants were streaked out on 5-FOA containing plates and after plasmid-shuffling spotted in 10-fold serial dilutions on YPD plates. Colony growth was monitored at the indicated temperatures and times. **b**, Cgr1 (amino acids 42-114) in the cryo-EM structure of the early Nog2 pre-60S particle (PDB: 3jct,[2]) in which the 5S RNP is in the non-rotated (immature) state. The positions of the *cgr1*ΔN51 truncation and the *cgr1*R108A R109A R110A point mutations are indicated. The 25S rRNA (light grey) and the 5S rRNA (orange) are displayed as surface models. H38 of the 25S rRNA (cyan), Cgr1 (red), Rpl5 (blue), Rpl11 (light blue), and Rsa4 (violet) are depicted.

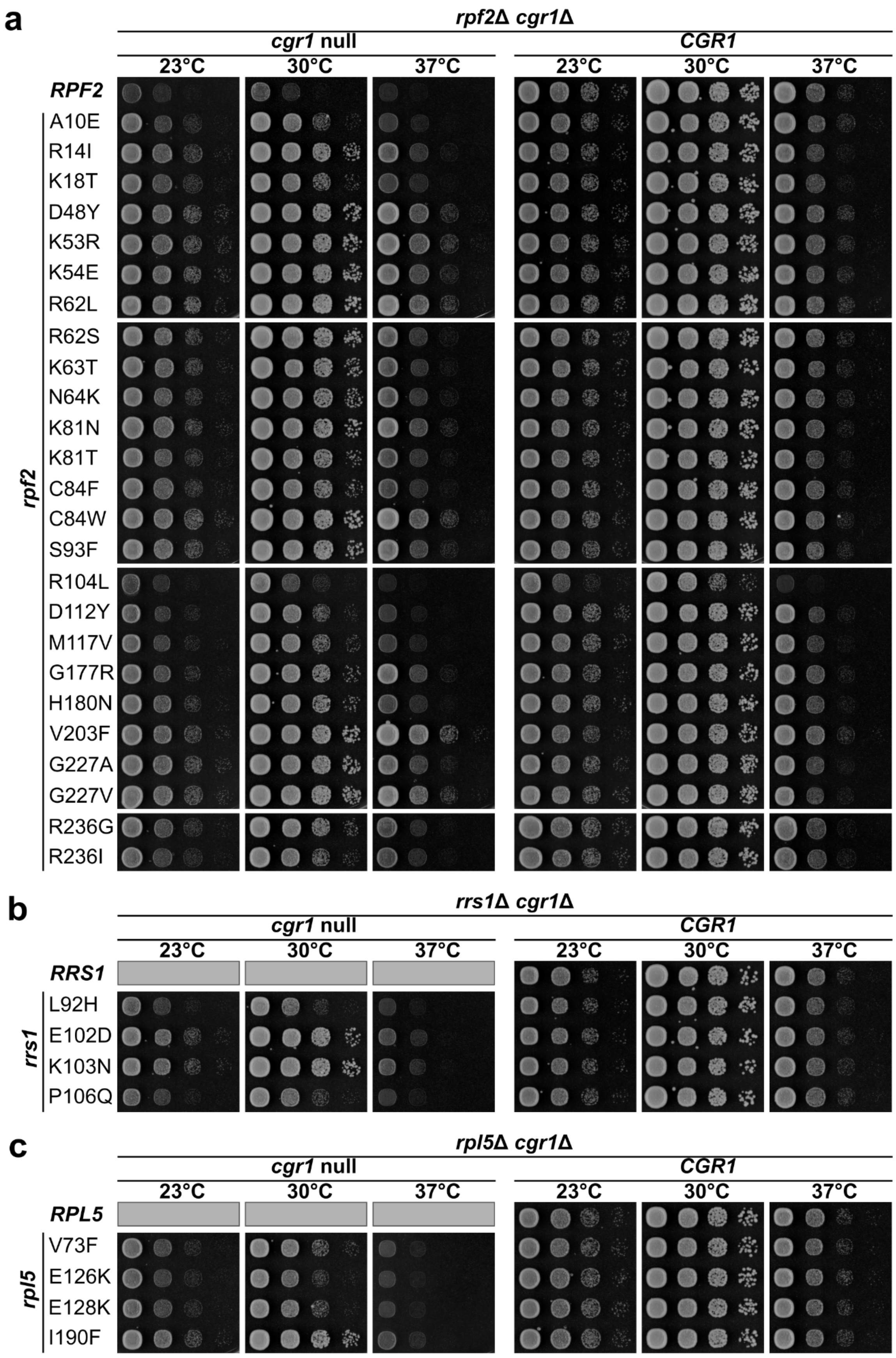
a
rpf2Δ cgr1Δ
cgr1 null
CGR1
23°C
30°C
37°C
RPF2
rpf2
A10E
R14I
K18T
D48Y
K53R
K54E
R62L
R62S
K63T
N64K
K81N
K81T
C84F
C84W
S93F
R104L
D112Y
M117V
G177R
H180N
V203F
G227A
G227V
R236G
R236I
b
rrs1Δ cgr1Δ
RRS1
rrs1
L92H
E102D
K103N
P106Q
c
rpl5Δ cgr1Δ
RPL5
rpl5
V73F
E126K
E128K
I190F

Supplementary Fig. 4 | Growth analyses of all the isolated *cgr1*Δ suppressor mutants. Suppressor alleles of the various *cgr1*Δ suppressor mutants were PCR-amplified from genomic DNA and cloned into plasmids under control of the respective endogenous promoter. DNA sequencing of the suppressor alleles identified the suppressor mutations. **a**–**c**, The indicated double-shuffle strains (*rpf2*Δ *cgr1*Δ, *rrs1*Δ *cgr1*Δ, *rpl5*Δ *cgr1*Δ) were co-transformed with plasmids harbouring the cloned *rpf2*, *rrs1* or *rpl5* suppressor alleles, or the respective wild-type alleles, combined with plasmids carrying wild-type *CGR1* or empty plasmid (*cgr1*Δ). After selection on SDC+FOA plates, representative progeny cells were spotted in 10-fold serial dilutions on YPD plates and growth was monitored after incubation for 40 h at the indicated temperatures. Note that co-transformants *RRS1*/empty vector and *RPL5*/empty vector did not grow on the initial SDC+FOA plates. The reason for this is not known, but it is possible that plasmid-borne *RRS1* and *RPL5* might enhance the already extreme slow-growth phenotype of *cgr1*Δ cells.

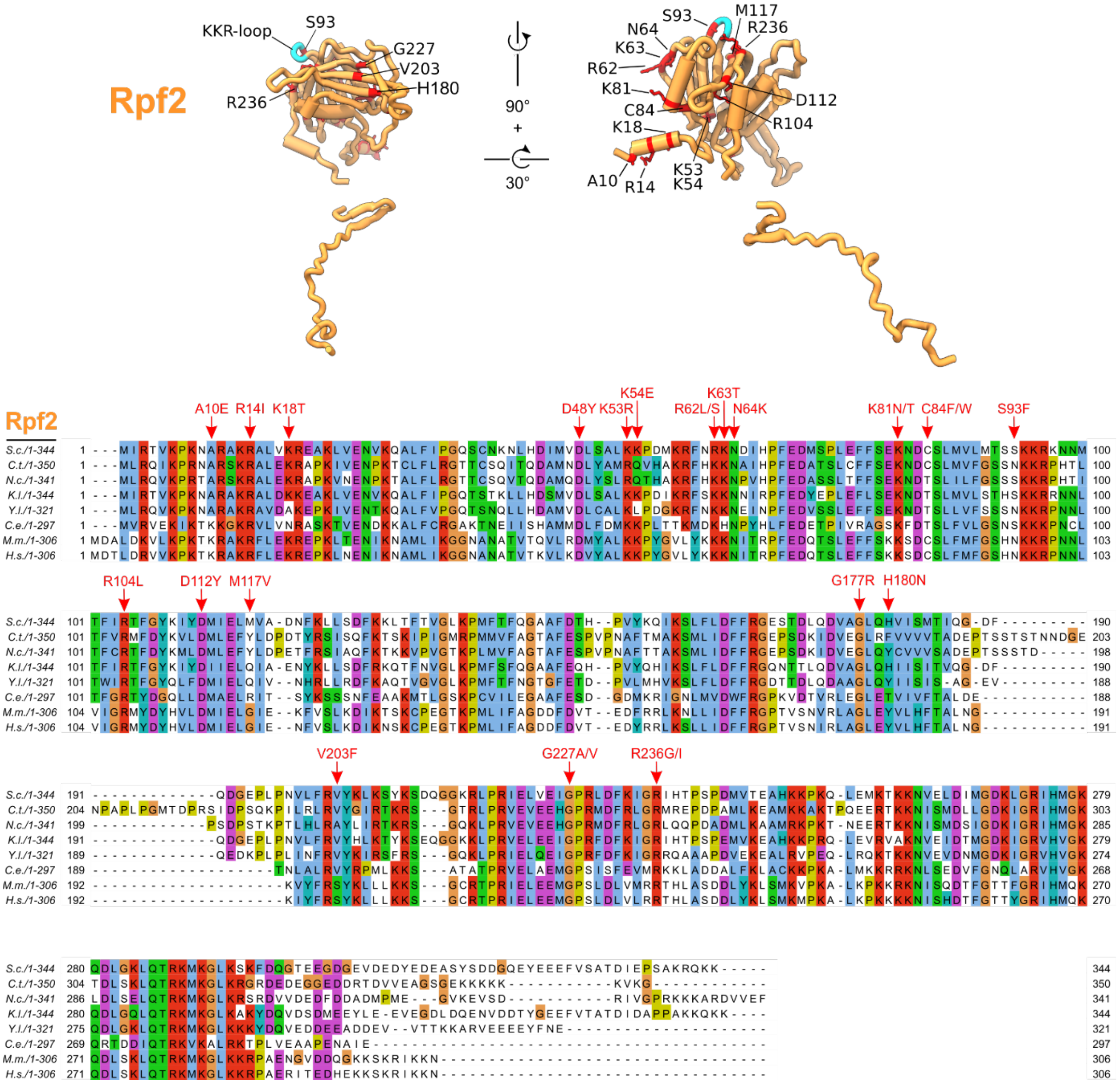

Supplementary Fig. 5 | Mapping of identified *cgr1*Δ suppressor mutations within Rpf2. Upper panel: residues in the Rpf2 cryo-EM structure (PDB: 3jct,[2]) that are mutated in *cgr1*Δ null suppressors are depicted in red. The KKR-loop (residues 94–96) is highlighted in cyan. Lower panel: multiple sequence alignment of Rpf2 orthologues from *Saccharomyces cerevisiae (S.c.)*, *Chaetomium thermophilum* (*C.t.*), *Neurospora crassa* (*N.c.*), *Kluyveromyces* lactis (*K.l.*), *Yarrowia lipolytica* (*Y.l.*), *Caenorhabditis elegans* (*C.e.*), *Mus musculus* (*M.m.*), and *Homo sapiens* (*H.s.*). The positions of the *cgr1*Δ suppressor mutations in the *S. cerevisiae* sequence are indicated by an arrow.

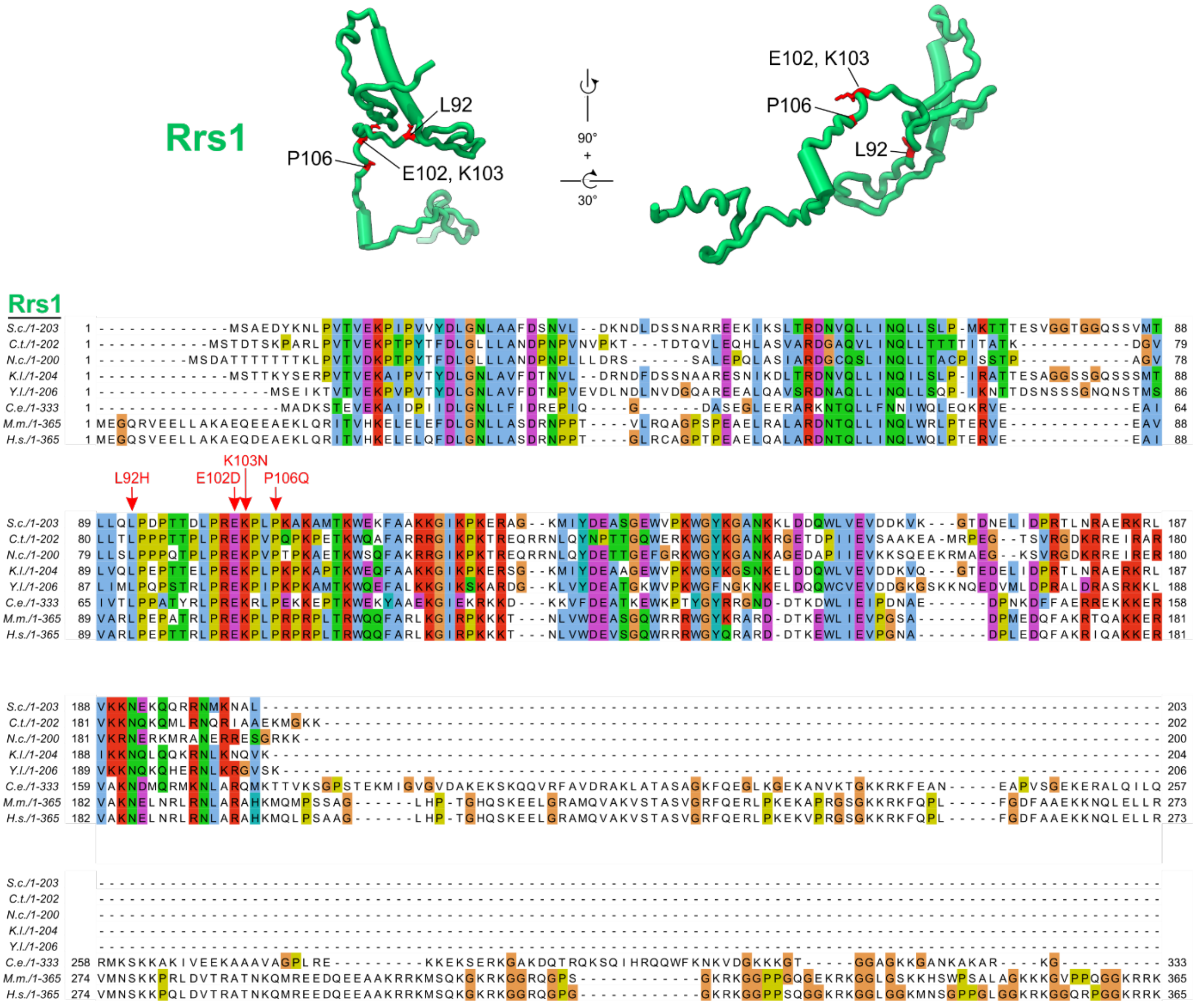

Supplementary Fig. 6 | Mapping of identified *cgr1*Δ suppressor mutations within Rrs1. Upper panel: residues in the Rrs1 cryo-EM structure (PDB: 3jct,[2]) that are mutated in *cgr1*Δ null suppressors are depicted in red. Lower panel: multiple sequence alignment of Rrs1 orthologues from *Saccharomyces cerevisiae* (*S.c.*), *Chaetomium thermophilum* (*C.t.*), *Neurospora crassa* (*N.c.*), *Kluyveromyces* lactis (*K.l.*), *Yarrowia lipolytica* (*Y.l.*), *Caenorhabditis elegans* (*C.e.*), *Mus musculus* (*M.m.*), and *Homo sapiens* (*H.s.*). The positions of the *cgr1*Δ suppressor mutations in the *S. cerevisiae* sequence are indicated by an arrow.

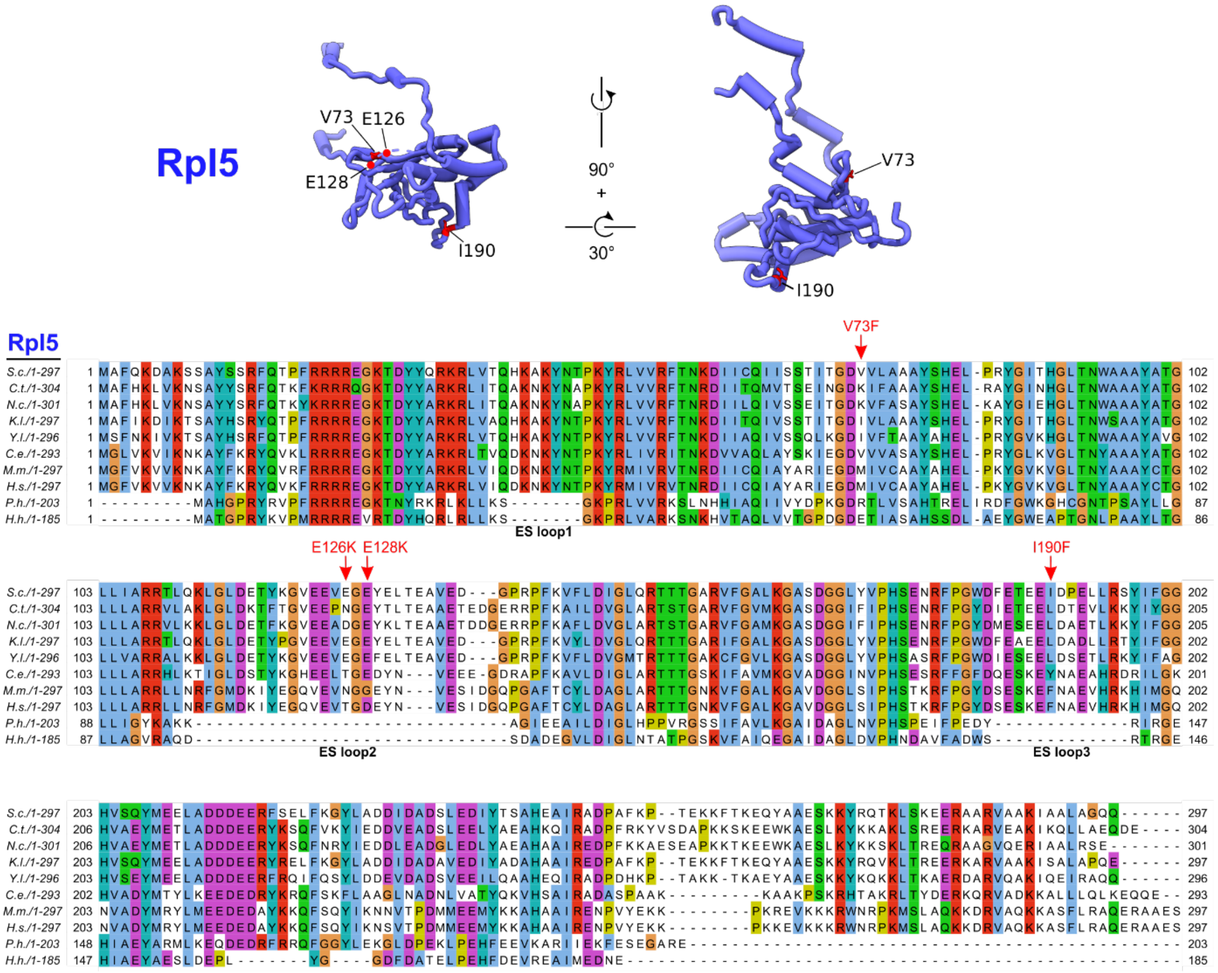

Supplementary Fig. 7 | Mapping of identified *cgr1*Δ suppressor mutations within Rpl5. Upper panel: residues in the Rpl5 cryo-EM structure (PDB: 3jct,[2]) that are mutated in *cgr1*Δ null suppressors are depicted in red. Lower panel: Multiple sequence alignment of Rpl5 orthologues from *Saccharomyces cerevisiae* (*S.c.*), *Chaetomium thermophilum* (*C.t.*), *Neurospora crassa* (*N.c.*), *Kluyveromyces* lactis (*K.l.*), *Yarrowia lipolytica* (*Y.l.*), *Caenorhabditis elegans* (*C.e.*), *Mus musculus* (*M.m.*), *Homo sapiens* (*H.s.*), *Pyrococcus horikoshii* (*P.h.*) and *Halobacterium hubeiense* (*H.h.*). Eukaryote specific (ES) loop sequences are depicted and the positions of the *cgr1*Δ suppressor mutations in the *S. cerevisiae* sequence are indicated by an arrow.

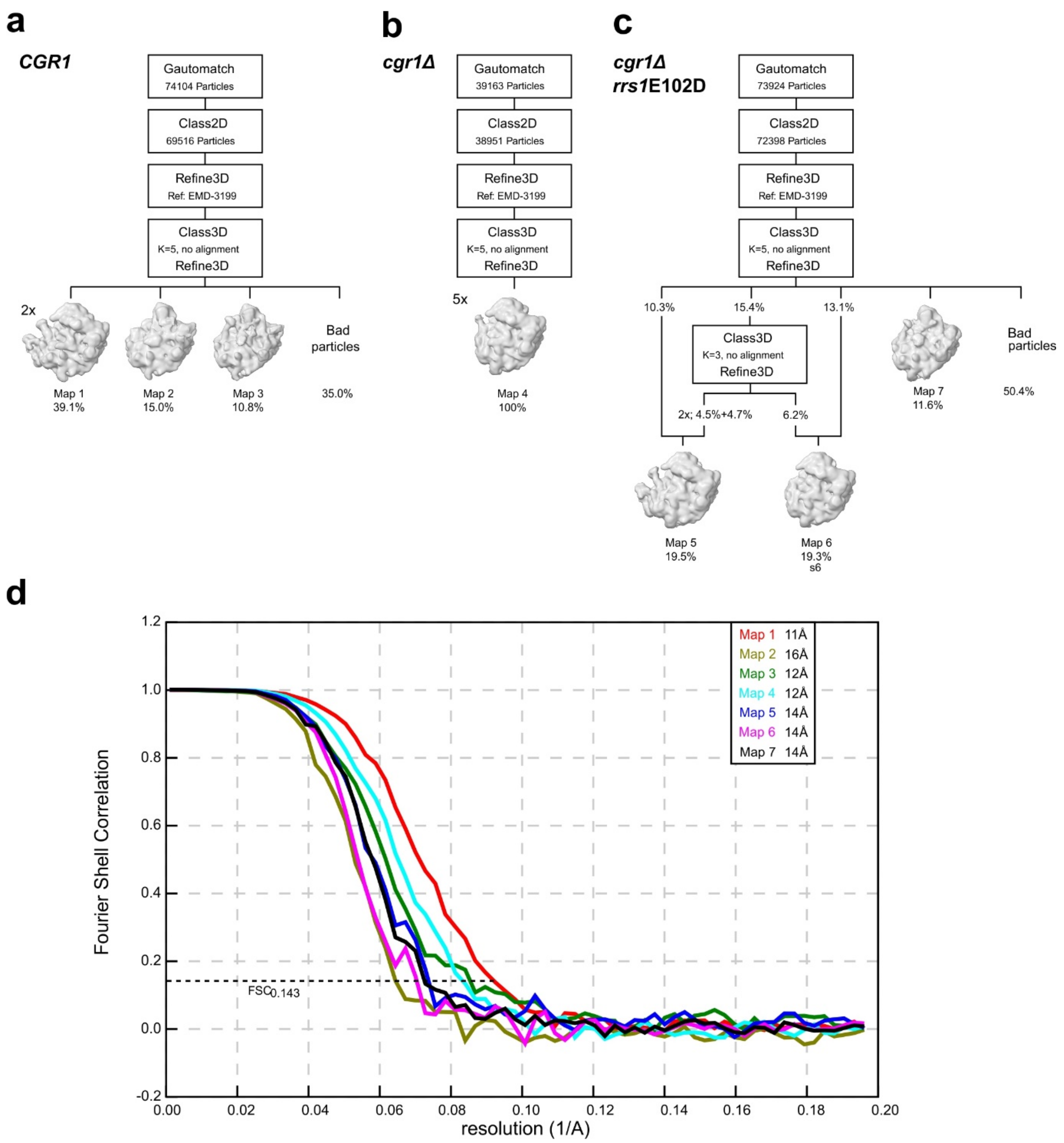

Supplementary Fig. 8 | Cryo-EM processing schemes and resolution estimation. a–c, Processing schemes of the three acquired cryo-EM datasets of *rrs1*Δ [YCplac111-*RRS1*] Arx1-FTpA Cgr1-HA-AID; -auxin (**a**), *cgr1*Δ depleted: *rrs1*Δ [YCplac111-*RRS1*] Arx1-FTpA Cgr1-HA-AID; +auxin (2h) (**b**) and *cgr1*Δ depleted in the presence of the *rrs1*E102D suppressor: *rrs1Δ* [YCplac111-*rrs1*E102D] Arx1-FTpA Cgr1-HA-AID; +auxin (2h) (**c**). **d**, Fourier shell correlation plots of Maps 1 – 7.

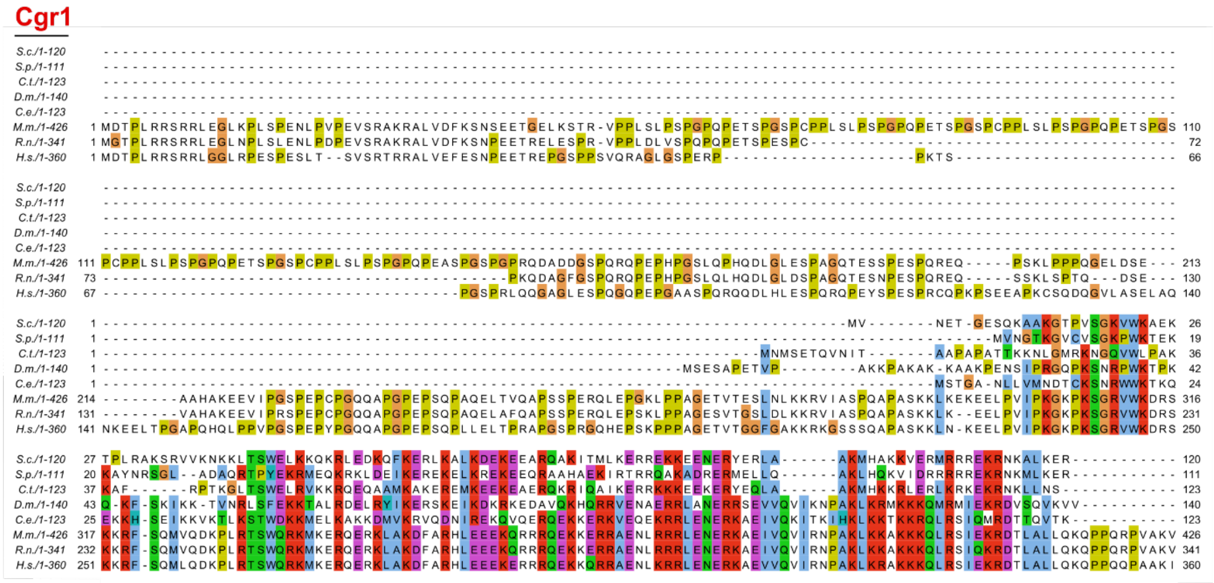

Supplementary Fig. 9 | Cgr1 is conserved between yeast and humans. Multiple sequence alignment of Cgr1 orthologues from *Saccharomyces cerevisiae* (*S.c.*), *Schizosaccharomyces pombe* (*S.p.*), *Chaetomium thermophilum* (*C.t.*), *Drosophila melanogaster* (*D.m.*), *Caenorhabditis elegans* (*C.e.*), *Mus musculus* (*M.m.*), *Rattus norvegicus* (*R.n.*), and *Homo sapiens* (*H.s.*). Note that mammalian Cgr1 orthologues carry a long proline-rich extension at the N-terminus.

Related to Figure 1e

overlay of membrane and chemiluminescence images

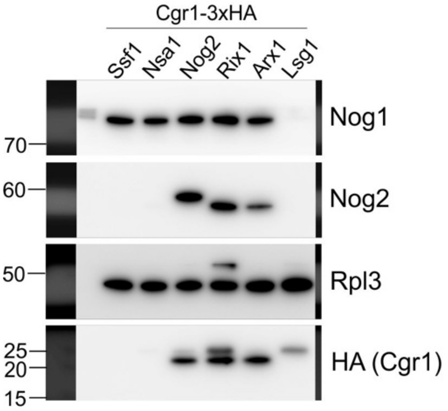

Related to Figure 2d

overlay of membrane and chemiluminescence images

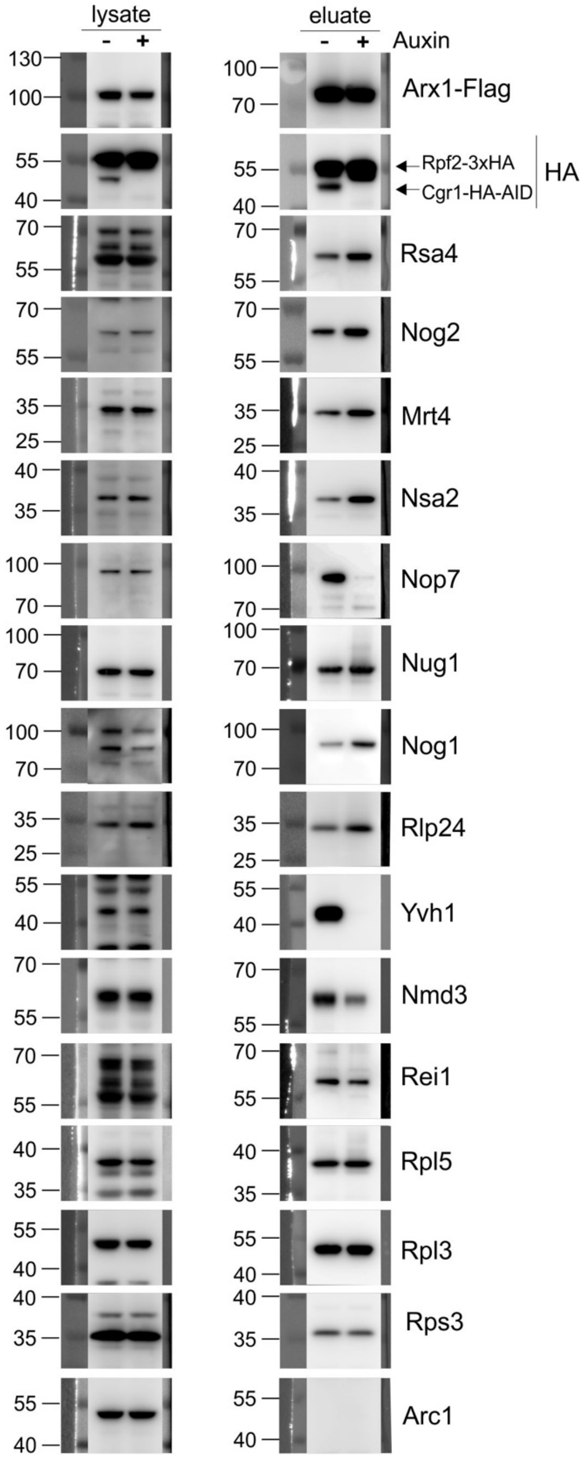

Related to Figure 5b

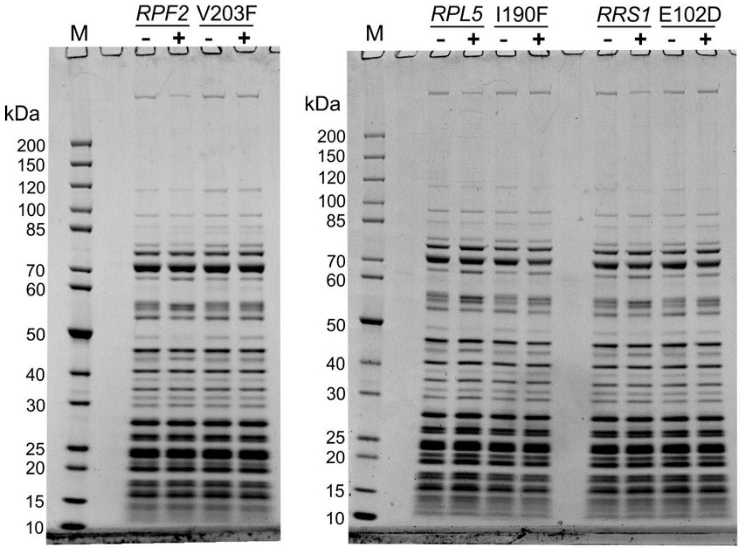

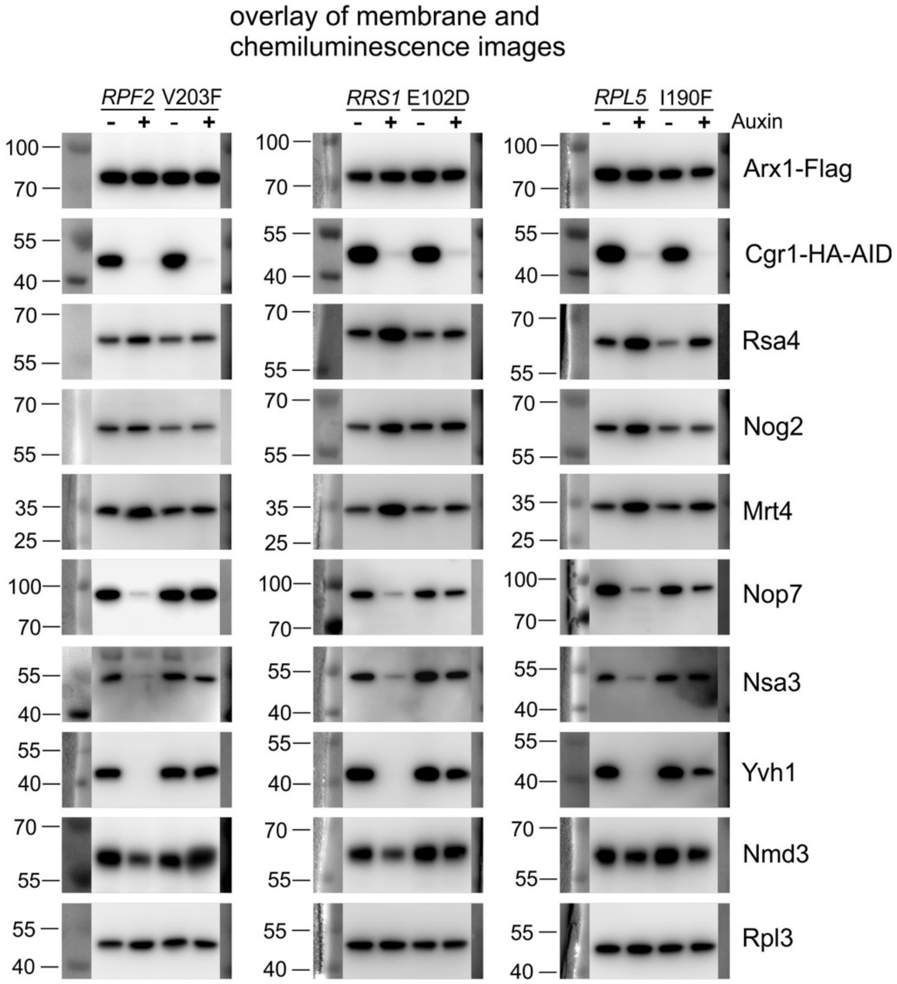

Supplementary Fig. 10 | Uncropped images of western blots and Coomassie-stained SDS-polyacrylamide gels.

Supplementary Table 1. **Cryo-EM data collection, refinement and validation statistics**

	Arx1-FTpA pre-60S particle Map_1 (EMDB-0218)	Arx1-FTpA pre-60S particle Map_2 (EMDB-0219)	Arx1-FTpA pre-60S particle Map_3 (EMDB-0220)	Arx1-FtpA pre-60S particle, cgr1 depleted Map_4 (EMDB-0221)	Arx1-FtpA pre-60S particle, cgr1 depleted, with suppressor Map_5 (EMDB-0222)	Arx1-FtpA pre-60S particle, cgr1 depleted, with suppressor Map_6 (EMDB-0223)	Arx1-FtpA pre-60S particle, cgr1 depleted, with suppressor Map_7 (EMDB-0224)
**Data collection and processing**							
Magnification	61200						
Voltage (kV)	120						
Electron exposure (e–/Å^2)	32						
Defocus range (μm)	0.8 – 3.2						
Pixel size (Å)	2.55						
Symmetry imposed	C1						
Initial particle images (no.)	74104			39163	73924		
Final particle images (no.)	27236	10380	7544	38951	14380	14112	8494
Map resolution (Å)	11 (FSC0.143)	16 (FSC0.143)	12 (FSC0.143)	12 (FSC0.143)	14 (FSC0.143)	14 (FSC0.143)	14 (FSC0.143)
FSC threshold							
Map resolution range (Å)	11-357	16-357	12-357	12-357	14-357	14-357	14-357

Supplementary Table 2. Yeast strains used in this study

Name	Genotype	Source
W303	*ade2-1, his3-11,15, leu2-3,112, trp1-1, ura3-1, can1-100*	Thomas and Rothstein,1989,[1]
TAP-Flag-*CGR1*	W303 *MAT*α natNT2::TAP-Flag-*CGR1*	this study
CGR1-FTpA	W303 *MAT*α *CGR1*-FTpA::natNT2	this study
CGR1 Shuffle	W303 *MAT*α *cgr1*::natNT2 [pRS316-*CGR1*]	this study
CGR1 Shuffle	W303 *MAT***a** *cgr1*::HIS3MX6 [pRS316-*CGR1*]	this study
CGR1-HA-AID	W303 *MAT*α *CGR1*-HA-AID::HIS3MX6 P.*ADH1*-Os*TIR1*-9xmyc::*TRP1*	this study
CGR1-HA-AID *ARX1*-FTpA	W303 *MAT*α *CGR1*-HA-AID::HIS3MX6 P.*ADH1*-Os*TIR1*-9xmyc::*TRP1* *ARX1*-FTpA::natNT2	this study
CGR1-HA-AID *ARX1*-FTpA *RPF2*-3xHA	W303 *MAT*α *CGR1*-HA-AID::HIS3MX6 P.*ADH1*-Os*TIR1*-9xmyc::*TRP1* *ARX1*-FTpA::natNT2 *RPF2*-3xHA::kanMX6	this study
SSF1-FTpA *CGR1*-3xHA	W303 *MAT*α *SSF1*- FTpA::natNT2 *CGR1*-3xHA::HIS3MX6	this study
NSA1-FTpA *CGR1*-3xHA	W303 *MAT*α *NSA1*- FTpA::natNT2 *CGR1*-3xHA::HIS3MX6	this study
RIX1-FTpA *CGR1*-3xHA	W303 *MAT*α *RIX1*- FTpA::natNT2 *CGR1*-3xHA::HIS3MX6	this study
ARX1-FTpA *CGR1*-3xHA	W303 *MAT*α *ARX1*- FTpA::natNT2 *CGR1*-3xHA::HIS3MX6	this study
LSG1-FTpA *CGR1*-3xHA	W303 *MAT*α *LSG1*- FTpA::natNT2 *CGR1*-3xHA::HIS3MX6	this study
CGR1 Shuffle *NSA2* Shuffle	W303 *cgr1*::HIS3MX6 [pRS316-*CGR1*] *nsa2*::kanMX6 [pRS316-*NSA2*]	this study
CGR1 Shuffle *NUG1* Shuffle	W303 *cgr1*::HIS3MX6 [pRS316-*CGR1*] *nug1*::kanMX6 [pRS316-*NUG1*]	this study
CGR1 Shuffle *RIX1* Shuffle	W303 *cgr1*::HIS3MX6 [pRS316-*CGR1*] *rix1*::natNT2 [pRS316-*RIX1*]	this study
CGR1 Shuffle *NOP7* Shuffle	W303 *cgr1*::HIS3MX6 [pRS316-*CGR1*] *nop7*::kanMX6 [pRS316-*NOP7*]	this study
CGR1 Shuffle *RPF2* Shuffle	W303 *MAT***a** *cgr1*::natNT2 [pRS316-*CGR1*] *rpf2*::HIS3MX6 [pRS316-*RPF2*]	this study
CGR1 Shuffle *RRS1* Shuffle	W303 *MAT***a** *cgr1*::natNT2 [pRS316-*CGR1*] *rrs1*::HIS3MX6 [pRS316-*RRS1*]	this study
CGR1 Shuffle *RPL5* Shuffle	W303 *MAT***a** *cgr1*::natNT2 [pRS316-*CGR1*] *rpl5*::HIS3MX6 [pRS316-*RPL5*]	this study
RPF2 Shuffle *CGR1*-HA-AID	W303 *MAT*α *rpf2*::hphNT1 [pRS316-*RPF2*] *CGR1*-HA-AID::HIS3MX6 P.*ADH1*-Os*TIR1*-9xmyc::*TRP1*	this study
RRS1 Shuffle *CGR1*-HA-AID	W303 *MAT*α *rrs1*::hphNT1 [pRS316-*RRS1*] *CGR1*-HA-AID::HIS3MX6 P.*ADH1*-Os*TIR1*-9xmyc::*TRP1*	this study

RPL5 Shuffle *CGR1*-HA-AID	W303 *MAT*α *rpl5*::hphNT1 [pRS316-*RPL5*] *CGR1*-HA-AID::HIS3MX6 P.*ADH1*-Os*TIR1*-9xmyc::*TRP1*	this study
RPF2 Shuffle *CGR1*-HA-AID *ARX1*-FTpA	W303 *MAT*α *rpf2*::hphNT1 [pRS316-*RPF2*] *CGR1*-HA-AID::HIS3MX6 P.*ADH1*-Os*TIR1*-9xmyc::*TRP1* *ARX1*-FTpA::natNT2	this study
RRS1 Shuffle *CGR1*-HA-AID *ARX1*-FTpA	W303 *MAT*α *rrs1*::hphNT1 [pRS316-*RRS1*] *CGR1*-HA-AID::HIS3MX6 P.*ADH1*-Os*TIR1*-9xmyc::*TRP1* *ARX1*-FTpA::natNT2	this study
RPL5 Shuffle *CGR1*-HA-AID *ARX1*-FTpA	W303 *MAT*α *rpl5*::hphNT1 [pRS316-*RPL5*] *CGR1*-HA-AID::HIS3MX6 P.*ADH1*-Os*TIR1*-9xmyc::*TRP1* *ARX1*-FTpA::natNT2	this study

Supplementary Table 3. Plasmids used in this study

Name	Relevant information	Source
YCplac111-eGFP-*CGR1*	CEN*, LEU2,* P*CGR1, CGR1*, N-terminal eGFP tag	this study
YCplac111-*CGR1*	CEN*, LEU2,* P*CGR1, CGR1*	this study
YCplac22-*CGR1*	CEN*, TRP1,* P*CGR1, CGR1*	this study
pRS316-*CGR1*	CEN*, URA3,* P*CGR1, CGR1*	this study
YCplac111-*cgr1*ΔN51	CEN*, LEU2,* P*CGR1, cgr1* aa 52-120	this study
YCplac22-*cgr1*ΔN51	CEN*, TRP1,* P*CGR1, cgr1* aa 52-120	this study
YCplac111- *cgr1*(RRR>AAA)	CEN*, LEU2,* P*CGR1, cgr1*R108A/R109A/R110A	this study
YCplac22- *cgr1*(RRR>AAA)	CEN*, TRP1,* P*CGR1, cgr1*R108A/R109A/R110A	this study
pRS314-*NSA2*	CEN*, TRP1,* P*NSA2, NSA2*	Bassler et al., 2014, [3]
pRS314-*nsa2-1*	CEN*, TRP1,* P*NSA2, nsa2-1*	Bassler et al., 2014, [3]
pRS314-*NUG1*	CEN*, TRP1,* P*NUG1, NUG1*	Bassler et al., 2001, [4]
pRS314-*nug1-1*	CEN*, TRP1,* P*NUG1, nug1-1*	Bassler et al., 2001, [4]
pRS315-*RIX1*	CEN*, LEU2,* P*RIX1, RIX1*	this study
pRS315-*rix1-1*	CEN*, LEU2,* P*RIX1, rix1-1*	Bassler et al., 2001, [4]
pRS314-*NOP7*	CEN*, TRP1,* P*NOP7, NOP7*	Du and Stillman, 2002, [5]
pRS314-*nop7-1*	CEN*, TRP1,* P*NOP7, nop7-1*	Du and Stillman, 2002, [5]
YCplac22-*GAL1-10-RPF2*	CEN, *TRP1*, P*GAL1-10*, *RPF2*	this study
YCplac22-*GAL1-10-RRS1*	CEN, *TRP1*, P*GAL1-10*, *RRS1*	this study
YCplac22-*GAL1-10-RPL5*	CEN, *TRP1*, P*GAL1-10*, *RPL5*	this study
YCplac22-*GAL1-10-RPL11*	CEN, *TRP1*, P*GAL1-10*, *RPL11*	this study
YCplac111-*RPF2*	CEN, *LEU2*, P*RPF2, RPF2*	this study
pRS316-*RPF2*	CEN*, URA3,* P*RPF2, RPF2*	this study
YCplac111-*rpf2*A10E	CEN, *LEU2*, P*RPF2, rpf2*A10E	this study

YCplac111-*rpf2*R14I	CEN, *LEU2*, P*RPF2, rpf2*R14I	this study
YCplac111-*rpf2*K18T	CEN, *LEU2*, P*RPF2, rpf2*K18T	this study
YCplac111-*rpf2*D48Y	CEN, *LEU2*, P*RPF2, rpf2*D48Y	this study
YCplac111-*rpf2*K53R	CEN, *LEU2*, P*RPF2, rpf2*K53R	this study
YCplac111-*rpf2*K54E	CEN, *LEU2*, P*RPF2, rpf2*K54E	this study
YCplac111-*rpf2*R62L	CEN, *LEU2*, P*RPF2, rpf2*R62L	this study
YCplac111-*rpf2*R62S	CEN, *LEU2*, P*RPF2, rpf2*R62S	this study
YCplac111-*rpf2*K63T	CEN, *LEU2*, P*RPF2, rpf2*K63T	this study
YCplac111-*rpf2*N64K	CEN, *LEU2*, P*RPF2, rpf2*N64K	this study
YCplac111-*rpf2*K81N	CEN, *LEU2*, P*RPF2, rpf2*K81N	this study
YCplac111-*rpf2*K81T	CEN, *LEU2*, P*RPF2, rpf2*K81T	this study
YCplac111-*rpf2*C84F	CEN, *LEU2*, P*RPF2, rpf2*C84F	this study
YCplac111-*rpf2*C84W	CEN, *LEU2*, P*RPF2, rpf2*C84W	this study
YCplac111-*rpf2*S93F	CEN, *LEU2*, P*RPF2, rpf2*S93F	this study
YCplac111-*rpf2*R104L	CEN, *LEU2*, P*RPF2, rpf2*R104L	this study
YCplac111-*rpf2*D112Y	CEN, *LEU2*, P*RPF2, rpf2*D112Y	this study
YCplac111-*rpf2*M117V	CEN, *LEU2*, P*RPF2, rpf2*M117V	this study
YCplac111-*rpf2*G177R	CEN, *LEU2*, P*RPF2, rpf2*G177R	this study
YCplac111-*rpf2*H180N	CEN, *LEU2*, P*RPF2, rpf2*H180N	this study
YCplac111-*rpf2*V203F	CEN, *LEU2*, P*RPF2, rpf2*V203F	this study
YCplac111-*rpf2*G227A	CEN, *LEU2*, P*RPF2, rpf2*G227A	this study
YCplac111-*rpf2*G227V	CEN, *LEU2*, P*RPF2, rpf2*G227V	this study
YCplac111-*rpf2*R236G	CEN, *LEU2*, P*RPF2, rpf2*R236G	this study
YCplac111-*rpf2*R236I	CEN, *LEU2*, P*RPF2, rpf2*R236I	this study
YCplac111-*RRS1*	CEN, *LEU2*, P*RRS1, RRS1*	this study
pRS316-*RRS1*	CEN*, URA3,* P*RRS1, RRS1*	this study
YCplac111-*rrs1*L92H	CEN, *LEU2*, P*RRS1, rrs1*L92H	this study

YCplac111-*rrs1*E102D	CEN, *LEU2*, P*RRS1, rrs1*E102D	this study
YCplac111-*rrs1*K103N	CEN, *LEU2*, P*RRS1, rrs1*K103N	this study
YCplac111-*rrs1*P106Q	CEN, *LEU2*, P*RRS1, rrs1*P106Q	this study
YCplac111-*RPL5*	CEN, *LEU2*, P*RPL5, RPL5*	this study
pRS316-*RPL5*	CEN*, URA3,* P*RPL5, RPL5*	this study
YCplac111-*rpl5*V73F	CEN, *LEU2*, P*RPL5, rpl5*V73F	this study
YCplac111-*rpl5*E126K	CEN, *LEU2*, P*RPL5, rpl5*E126K	this study
YCplac111-*rpl5*E128K	CEN, *LEU2*, P*RPL5, rpl5*E128K	this study
YCplac111-*rpl5*I190F	CEN, *LEU2*, P*RPL5, rpl5*I190F	this study
pRS316-mRFP-*NOP1*-*RPL25*-eGFP	CEN, *URA3*, P*NOP1*, *NOP1*, N-terminal mRFP tag, P*RPL25*, *RPL25*, C-terminal eGFP tag	Ulbrich et al., 2009, [6]
pRS316- mRFP-*NOP1*-*RPS3*-eGFP	CEN, *URA3*, P*NOP1*, *NOP1*, N-terminal mRFP tag, P*RPS3*, *RPS3*, C-terminal eGFP tag	Ulbrich et al., 2009, [6]

P denotes promoter

Supplementary References

1. Thomas, B. J. & Rothstein, R. Elevated recombination rates in transcriptionally active DNA. *Cell* **56,** 619–630 (1989).
2. Wu, S. *et al.* Diverse roles of assembly factors revealed by structures of late nuclear pre-60S ribosomes. *Nature* **534,** 133–137 (2016).
3. Baßler, J. *et al.* A network of assembly factors is involved in remodeling rRNA elements during preribosome maturation. *J. Cell Biol.* **207,** 481–498 (2014).
4. Bassler, J. *et al.* Identification of a 60S preribosomal particle that is closely linked to nuclear export. *Mol. Cell* **8,** 517–529 (2001).
5. Du, Y.-C. N. & Stillman, B. Yph1p, an ORC-interacting protein: potential links between cell proliferation control, DNA replication, and ribosome biogenesis. *Cell* **109,** 835–848 (2002).
6. Ulbrich, C. *et al.* Mechanochemical removal of ribosome biogenesis factors from nascent 60S ribosomal subunits. *Cell* **138,** 911–922 (2009).
